# Initial Amino Acid Intake Influences Phosphorus and Calcium Homeostasis in Preterm Infants – It Is Time to Change the Composition of the Early Parenteral Nutrition

**DOI:** 10.1371/journal.pone.0072880

**Published:** 2013-08-15

**Authors:** Francesco Bonsante, Silvia Iacobelli, Giuseppe Latorre, Jacques Rigo, Claudio De Felice, Pierre Yves Robillard, Jean Bernard Gouyon

**Affiliations:** 1 Néonatologie et Réanimation Pédiatrique et Néonatale, Centre Hospitalier Universitaire de la Réunion, France; 2 Centre d’Etudes Perinatales de l’Océan Indien, La Réunion, France; 3 NICU and Neonatology, Miulli Hospital, Acquaviva delle Fonti, Italy; 4 Department of Paediatrics, Neonatal Unit, University of Liege, Liege, Belgium; 5 NICU and Neonatology, University Hospital, Policlinico le Scotte, Siena, Italy; INRA, France

## Abstract

**Background:**

Early aggressive parenteral nutrition (PN), consisting of caloric and nitrogen intake soon after birth, is currently proposed for the premature baby. Some electrolyte disturbances, such as hypophosphatemia and hypercalcemia, considered unusual in early life, were recently described while using this PN approach. We hypothesize that, due to its impact on cell metabolism, the initial amino acid (AA) amount may specifically influence the metabolism of phosphorus, and consequently of calcium. We aim to evaluate the influence of AA intake on calcium-phosphorus metabolism, and to create a calculation tool to estimate phosphorus needs.

**Methods:**

Prospective observational study. Phosphate and calcium plasma concentrations and calcium balance were evaluated daily during the first week of life in very preterm infants, and their relationship with nutrition was studied. For this purpose, infants were divided into three groups: high, medium and low AA intake (HAA, MAA, LAA). A calculation formula to assess phosphorus needs was elaborated, with a theoretical model based on AA and calcium intake, and the cumulative deficit of phosphate intake was estimated.

**Results:**

154 infants were included. Hypophosphatemia (12.5%) and hypercalcemia (9.8%) were more frequent in the HAA than in the MAA (4.6% and 4.8%) and in the LAA group (0% and 1.9%); both p<0.001.

**Discussion:**

Calcium-phosphorus homeostasis was influenced by the early AA intake. We propose to consider phosphorus and calcium imbalances as being part of a syndrome, related to incomplete provision of nutrients after the abrupt discontinuation of the placental nutrition at birth (PI-ReFeeding syndrome).

We provide a simple tool to calculate the optimal phosphate intake. The early introduction of AA in the PN soon after birth might be completed by an early intake of phosphorus, since AA and phosphorus are (along with potassium) the main determinants of cellular growth.

## Introduction

Recent nutrition guidelines recommend early introduction of “aggressive” parenteral nutrition (PN) for the preterm baby, in order to avoid cellular catabolism and to promote extra-uterine growth [1]-[4].

There is some current evidence that water and electrolyte balance, along with micronutrient homeostasis, may be influenced by early aggressive PN [5,6]. In previous investigations we have shown that a higher early Amino Acid (AA) intake may prevent non-oliguric hyperkalemia in very low birth weight infants by inhibiting cellular catabolism and promoting growth [7].

The metabolism of phosphorus and its plasma concentration may also be influenced by this new nutritional approach. Phosphate content is very abundant in the cell, as it represents the main anion in the intracellular space and it enters into the composition of the nucleic acids, the ATP and the cell membrane. The phosphate role in bone development and mineralization, but also in cellular growth, may cause dramatic variations in its serum concentration during early aggressive PN.

Recent clinical observations support this hypothesis. Mizumoto et al. [8] have observed a severe condition of hypophosphatemia and hypokalemia triggered by early aggressive PN in an extremely low birthweight infant (ELBWI). In a small retrospective cohort, Ichikawa et al. have shown that higher parenteral AA administration facilitated a condition of hypophosphatemia and hypercalcemia in ELBWI at the end of the first week of life [9]. In both studies the plasma phosphate levels in infants receiving PN were inversely associated to the AA intake. One study published by Moltu and colleagues in 2012 has further shown that enhanced feeding in very low birth weight infants may induce electrolyte imbalances (hypokalemia, hypophosphatemia and hypercalcemia) during the first week of life [10].

Indeed, phosphate concentration disturbances will also induce variations in calcium plasma levels and urinary excretion, due to the metabolic interactions of these two micronutrients during the bone resorption and formation process.

The present study is part of a prospective investigation carried out with the aim of assessing the relationship between the fluid and electrolyte homeostasis and the nutritional intake in a cohort of premature infants, as already described [11]. Some information about the influence of protein and caloric intake on fluid compartments and sodium and potassium balances has already been published in a previous paper [11].

The aims of this study are the following:

Firstly, to evaluate the influence of early nutrition on calcium and phosphate homeostasis in preterm infants;

Secondly, to create a simple calculation tool, in order to estimate the optimal amount of phosphorus to be added in the PN bags, according to the administered AA intake.

## Methods

### Design and Study Population

This was a prospective study was conducted from June 1, 2006 to September 30, 2007. All the infants born below 33 weeks of gestational age (GA) and hospitalized within 6 hours of life in the Neonatal Intensive Care Unit of the Dijon University Hospital were eligible. Infants with major congenital anomalies were excluded from the study.

### Ethics Statement

The study was conducted according to the principles of the declaration of Helsinki, and the study protocol was approved by the regional Ethics Committee (Comité de Protection des Personnes “Est-I”, Faculté de Médecine, BP 87900, 21079 Dijon Cedex). Informed, signed parental consent was obtained. The study was entirely supported by the institution.

### Nutritional Prescription

PN on central venous line or on peripheral venous line was administered according to clinical decision. In both cases, PN was administered by individualized formulations prepared into the unit or by standardized batch-produced bags, as already shown in a previous report [7]. In case of partial PN the intravenous nutritional intake was limited by the solution osmolarity.

Minimal enteral feeding by human milk was started on day one of life, and continued for at least 4 days in babies having total PN. When partial PN was given, enteral nutrition was started on day one at 20 ml/kg/day and daily increased over the week.

### Amino Acid, Calcium (Ca) and Phosphorus (P) intakes

AA intake (Primene 10%, Baxter) was started on day one and incremented daily up to 3.5 g/kg at the end of the first week in the standardized procedure. When PN was individualized, the initial amount and the rate of AA increase were decided by the prescribing physician, based on a written protocol available in the unit which suggested the day of life for initiation of each nutrient and energy intakes.

Ca infusion (Ca gluconate 10%, Aguettant) was started on the 1^st^ day of life at 40-50 mg/kg/d. P infusion (Phocytan, Aguettant) was started on the 2^nd^ or the 3^rd^ day, with wide variations among the infants, depending on the prescribing physician.

### Data Collection

Plasma sodium, potassium, Ca, P, total carbon dioxide, urea and creatinine were determined daily for 7 days. The first blood sample was obtained at around 12 hours of life. Daily diuresis was calculated and a consecutive 8-hour urine collection, starting 4 hours before the blood sampling, was performed using a plastic bag. Urine was analysed for sodium, potassium, Ca, urea and creatinine concentrations. Urine escaping the bag was quantified by weighing nappies. Fluid, energy and nutrient intakes were recorded daily. Oral human milk intakes were considered in fluid and nutrient intake calculations. Plasma and urine electrolytes were measured by an Ortho Clinical Diagnostic analyser (Rochester, NY, USA), which uses direct potentiometry. Ca balance was expressed in mg/kg/day and calculated as the difference between daily intake and daily urinary excretion.

### Statistics

First, the association of calcemia, phosphatemia and Ca balance with perinatal variables and energy, AA, Ca and P intake, was explored using one-factor analysis of variance. The perinatal variables entered into the model were: birth weight, gestational age, gender, small for gestational age, antenatal corticosteroids, caesarean section, postnatal age and respiratory distress syndrome. Variables significant at a P-value 0.10 at the univariate analysis were entered into a backward selection analysis of variance. Then, the infants were divided into three groups, according to the mean level of AA intake during the week: low AA intake: <1.5 g/kg/day (LAA); medium AA intake: 1.5-2 g/kg/d (MAA); high AA intake: >2 g/kg/day (HAA). Calcemia, phosphatemia and Ca balance were compared within the three groups. The crude risk for Ca and P perturbation was also evaluated. Data from continuous variables were expressed as mean ± SD. Differences among groups were studied using analysis of variance test, and results were adjusted for GA with analysis of covariance test. Linear regression procedures were used to correlate the cumulative calculated deficit in P intake with Ca and P plasma levels. Statistical tests were performed by SAS software 8.2 (SAS Institute Inc., Cary, NY, USA), results were considered significant at a 5% level.

### Modelling of the optimal phosphorus intake

A theoretical model for P need was elaborated as follows.

1. Estimated Ratio of Ca and P mass in bone, based on hydroxyapatite composition: P (mg) = Ca (mg) / 2.15 [12] 

2. Estimated Ratio of Nitrogen (AA) and P in the rapid growing cell: P (mg) = AA (g) * 12.3 [13]-[16] 

3. Estimated minimal AA intake to obtain a positive nitrogen balance: AA = 1.3 g/kg/d [1,2,11]

4. Estimated AA retention: AA = 80% of AA intake (exceeding the minimal AA intake)

Calculation Formula: P need = Ca intake/2.15 + (AA intake -1.3) * 0.8 * 12.3

P need is expressed in mg/kg/d; Ca intake is expressed in mg/kg/d; AA intake is expressed in g/kg/d.

The formula was based on total intakes. It does not take in account the enteral absorption rate of nutrients.

#### Testing the Model

The estimated P need was calculated for each day of the first week. Then we evaluated the daily P deficit by subtracting the real intake of P from the theoretical need. The cumulative deficit of P intake from birth was then calculated, and correlated to the Ca and P plasma levels. A second linear regression analysis was performed adjusting the daily plasma levels of Ca and P for their level on the first day of life in each infant.

## Results

One hundred and fifty-four infants were included. A total of 813 Ca and 436 P plasma determinations were performed. In total, 465 Ca urine collections were obtained.

Table 1 shows the multivariate analysis. The main independent factor influencing calcemia and phosphatemia was AA intake. Also phosphorus intake was an important independent factor for both Ca and P plasma levels.

**Table 1:** Multivariate analysis for plasma phosphate and calcium concentration and calcium balance.

		**β-coefficient**	**r-partial**	**P-value**
**Calcemia (r^2^ 0.38)**				
	Amino acid intake	0.038	0.29	<0.001
	Postnatal age	0.060	0.22	<0.001
	Phosphorus intake	-0.002	-0.18	<0.001
	Gestational age	0.015	0.14	<0.001
	Caloric Intake	-0.001	-0.07	0.04
**Phosphatemia (r^2^ 0.24)**				
	Amino acid intake	-0.090	-0.40	<0.001
	Phosphorus intake	0.004	0.20	<0.001
	Caloric Intake	0.002	0.14	0.002
	Postnatal age	-0.069	-0.13	0.006
**Calcium balance (r^2^ 0.37)**				
	Calcium intake	0.28	0.53	<0.001
	Postnatal age	-13.5	-0.51	<0.001
	Amino acid intake	-1.63	-0.28	<0.001
	Diuresis	-0.90	-0.14	0.002
	Phosphorus intake	0.05	0.10	0.03

Antenatal, postnatal characteristics and nutritional intake for the three groups are summarized in Table 2. The HAA group was similar to the MAA group with regard to BW and GA, but differed from the LAA group. AA intake was different by definition among the groups, as was energy intake. Ca and P intake were not much different in the groups.

**Table 2:** Characteristics, nutritional intake and theoretical phosphate need for 154 infants divided into 3 groups, according with their mean amino acid intake.

		**Groups (number of patients)**			
		**LAA (48)**	**MAA (53)**	**HAA (53)**	**P**
**Characteristics**					
BW (g)	mean ± SD	1595 ± 284	1210 ± 295*	1143± 332*	<0.001
GA (weeks)	mean ± SD	31.2 ± 1.4	29.2 ± 1.9*	29.2± 1.6*	<0.001
SGA	%	12	13	17	n.s.
Antenatal steroids	%	89	74	83	n.s.
Caesarean section	%	76	77	85	n.s.
RDS requiring surfactant	%	66	83	85	<0.05
Oxygen dependency at 36 weeks	%	4.3	13	19*	n.s.
Necrotizing enterocolitis	%	2.1	1.9	1.9	n.s.
Several abnormal cerebral ultrasound^a^	%	2.1	1.9	1.9	n.s.
**Mean daily nutritional intakes**					
Amino acids (g/kg/d)	mean ± SD	1.2 ± 0.6	1.8 ± 0.7*	2.3 ± 0.8*^§^	<0.001
Energy (Kcal/kg/d)	mean ± SD	55 ± 21	58 ± 22*	65 ± 25*^§^	<0.001
Calcium (mg/kg/d)	mean ± SD	46.7 ± 13.1	49.3 ± 8.6	50.3 ± 7.7	n.s.
Phosphate (mg/kg/d)	mean ± SD	15.8 ± 13.6	19.9 ± 13.9	21.4 ± 14.6*	0.04
Enteral feeding (mL/kg/d)	mean	39.0	16.5*	11.1*^§^	<0.001
**Theoretical calculations in phosphate intakes**					
Estimated phosphate need (mg/kg/d)	mean ± SD	20.7 ± 12.9	27.8 ± 12.7*	33.2 ± 14.0*^§^	<0.001
Deficit of phosphate intake (mg/kg/d)	mean ± SD	4.9 ± 13.4	7.9 ± 10.7*	11.8 ± 9.6*^§^	<0.001

LAA group: <1.5 g/kg/day of mean AA intake; MAA group: 1.5-2 g/kg/d of mean AA intake; HAA group: >2 g/kg/day of mean AA intake (BW). birth weight ; ( GA ) gestational age ; ( SGA ) Small for Gestational Age ; ( RDS ) Respiratory Distress Syndrome . ^a^ Intraventricular haemorrhage grade 3 or 4 and/or periventricular leukomalacia . *p<0.05 versus LAA group . §p<0.05 versus MAA group.

The group analysis confirmed the influence of the AA intake on the homeostasis of Ca. Calcemia was significantly higher in the HAA group as was the incidence of severe hypercalcemia (Ca > 2.8 mmol/L). Calcium urine output was very important in the HAA group by the end of the week. Despite these high levels of calciuria, the Ca balance remained positive at any time for all the groups. Phosphatemia was strongly influenced by early nutrition, with very low plasma levels in the HAA group and a significant risk of hypophosphatemia (P < 1 mmol/L) (Table 3).

**Table 3:** Calcium and phosphate homeostasis among groups.

		**Groups (number of patients)**				
		**LAA (48)**	**MAA (53)**	**HAA (53)**	**ANOVA**	**ANCOVA**
**Phosphatemia (mmol/L)**	mean ± SD	1.67 ± 0.32	1.49 ± 0.34*	1.38± 0.33*^§^	<0.001	<0.001
**Calcemia (mmol/L)**	mean ± SD	2.37 ± 0.21	2.41 ± 0.22*	2.41 ± 0.22*	<0.001	<0.001
**Calciuria (mmol/kg/d)**	mean ± SD	0.06 ± 0.06	0.12 ± 0.15*	0.19 ± 0.22*^§^	<0.001	<0.001
**Calcium Balance (mg/kg/d)**	mean ± SD	45.6 ± 10.2	44.7 ± 10.9	42.4 ± 12.9	n.s.	
**Severe hypophosphatemia**	N of cases (%)	0/136 (0.0)	6/130 (4.6)*	21/168 (12.5)*	<0.001	
	N of infants (%)	0/48 (0.0)	3/53 (5.7)	10/53 (18.9)*	<0.001	
**Severe hypercalcemia**	N of cases (%)	4/213 (1.9)	12/248 (4.8)*	29/295 (9.8)*	<0.001	
	N of infants (%)	3/48 (6.2)	8/53 (15.1)	16/53 (30.2)*	0.05	

LAA group: <1.5 g/kg/day of mean AA intake; MAA group: 1.5-2 g/kg/d of mean AA intake; HAA group: >2 g/kg/day of mean AA intake. Severe hypophosphatemia is defined to be < 1 mmol/L , severe hypercalcemia is defined as > 2.8 mmol/L . *p<0.05 versus LAA group . §p<0.05 versus MAA group.

**Figure 1:**
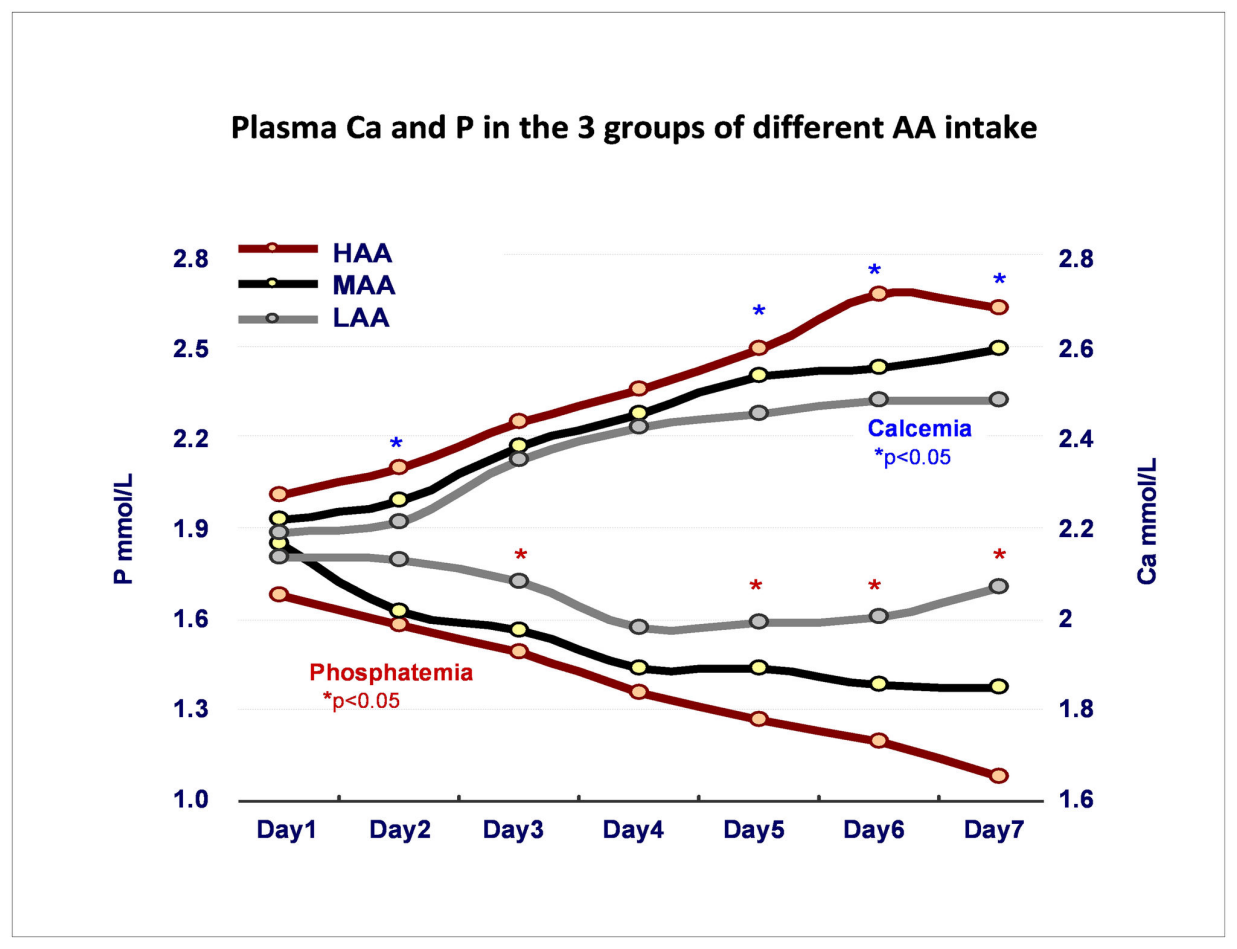
Plasma concentrations of calcium and phosphate in the 3 groups with different amino acid intake. LAA group: <1.5 g/kg/day of mean AA intake; MAA group: 1.5-2 g/kg/d of mean AA intake; HAA group: >2 g/kg/day of mean AA intake.

**Figure 2:**
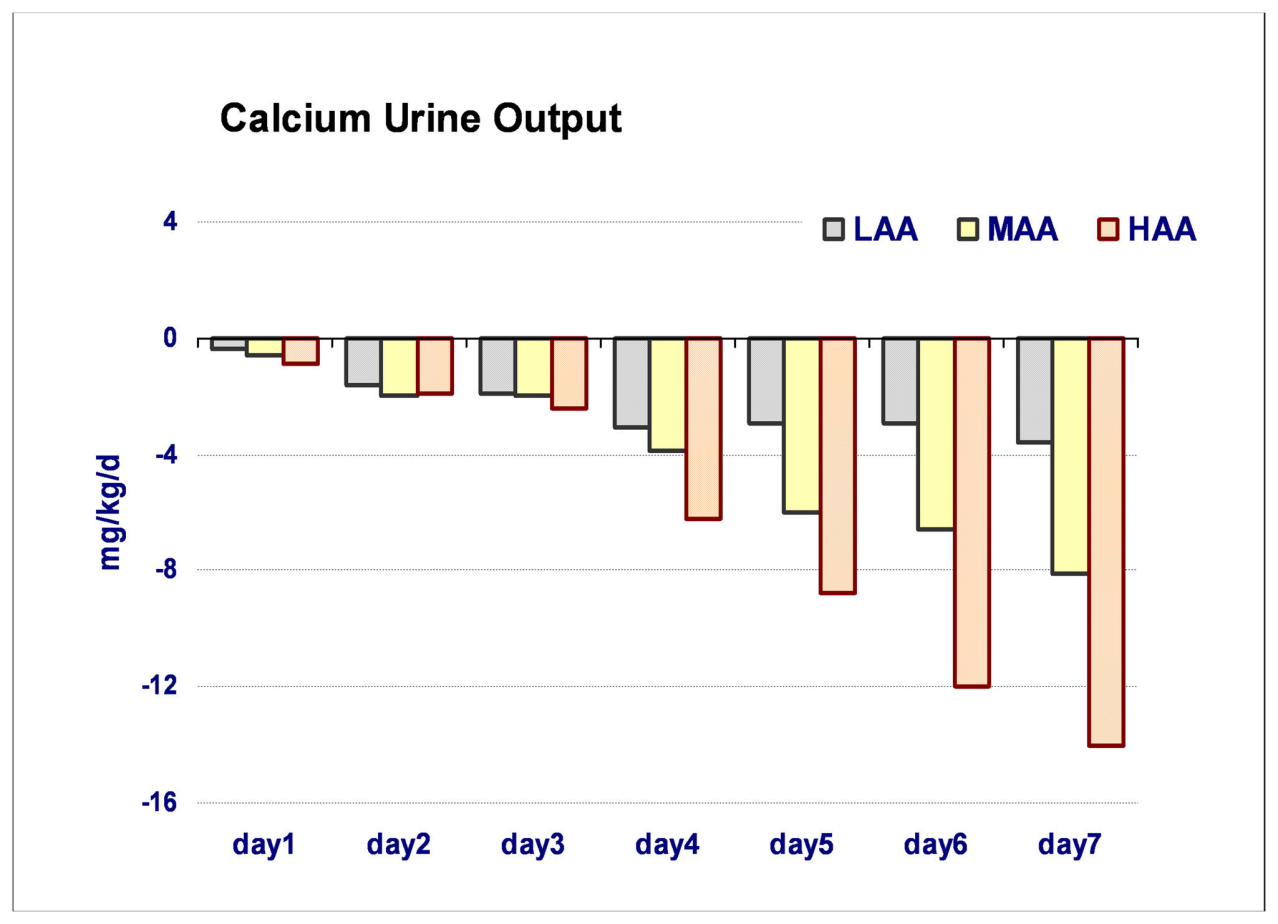
Calcium urine output in the 3 groups with different amino acid intake. LAA group: <1.5 g/kg/day of mean AA intake; MAA group: 1.5-2 g/kg/d of mean AA intake; HAA group: >2 g/kg/day of mean AA intake.

Figure 1 shows the day-by-day evolution of plasma Ca and P, with a clear inverse relationship between the two ions. Figure 2 presents the daily urine output of calcium.

**Figure 3:**
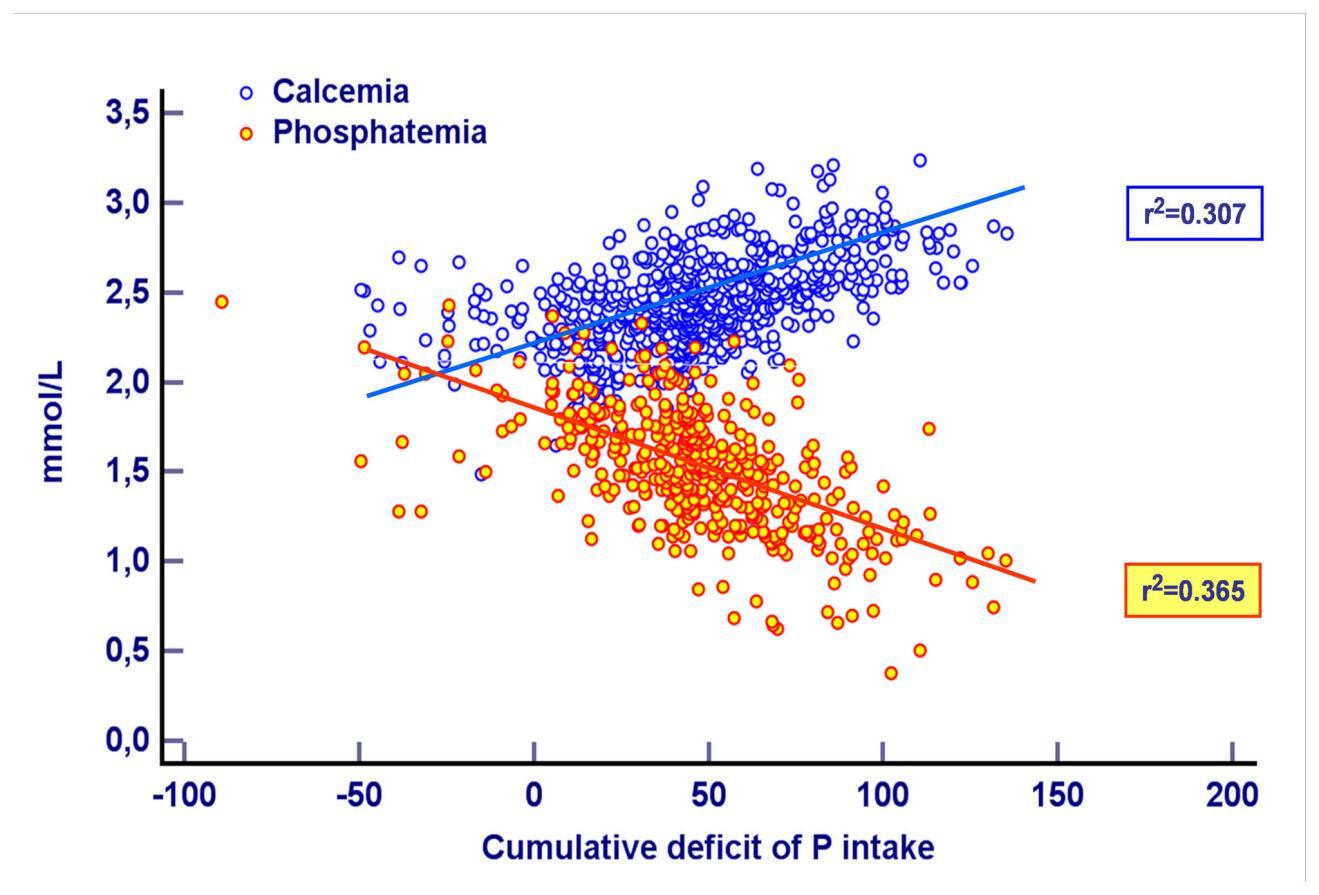
Linear regression function showing the relationship between the cumulative deficit of phosphorus intake and the plasma concentrations of calcium and phosphate.

Table 2 shows the estimated P need and P deficit for the 3 groups. The results of linear regression procedures between the cumulative estimated P deficit and calcium and phosphate plasma levels are expressed in Table 4 and graphically shown in Figure 3.

**Table 4:** Linear regression between cumulative estimated deficit of phosphorus intake and calcium and phosphate plasma level.

**Linear regression procedure**	**r^2^**	**F-ratio**	**P**
Calcemia vs. cumulative deficit of P intake	0.210	213	<0.001
Adjusted calcemia vs. cumulative deficit of P intake*	0.307	357	<0.001
Phosphatemia vs. cumulative deficit of P intake	0.173	90	<0.001
Adjusted phosphatemia vs. cumulative deficit of P intake**	0.365	257	<0.001

* Adjusted on first day calcium plasma level. ** Adjusted on first day phosphate plasma level.

x

## Discussion

The modern approach to nutrition of the premature infant is based on starting feeding as soon as possible after birth, supplying a parenteral continuous flow of AA and energy (early “aggressive” parenteral nutrition). According to some of the latest studies it appears clear, however, that this new approach can be associated with some important metabolic disturbances in the first days of life [17].

In particular, the early feeding strongly influences the metabolism of potassium, phosphorus and calcium, defining a syndrome that somehow simulates what happens in re-feeding conditions after intense nutritional deprivations, such as in Kwashiorkor, and in extreme bodybuilding [18,19].

For this reason, Mizumoto also used the definition of re-feeding syndrome for this condition of the premature infant [8].

The disturbances that we observe are no real surprise for the researcher. In fact, potassium and phosphorus are the main ions present in the cytoplasm, respectively with positive and negative charge. In addition, the phosphorus is a significant component of nucleic acids, ATP and membrane phospholipids. The provision of adequate amounts of nitrogen, potassium and phosphorus is the condition required to obtain a rapid cell growth whether in bacteria or in eukaryotic cells of both plants and animals. In addition, the variability of the ratios among these nutrients will be able to modulate the quality of growth [20].

In the human newborn, as in animals, rapidly growing cells will need large amounts of AA and potassium, so we can estimate that the needs of this cation are closely linked to the AA supply. In a previous report we showed that the balance of potassium closely parallels that of nitrogen [11].

When considering the metabolism of phosphorus, it is important to consider that it enters into the constitution of the soft tissues. The phosphorus to nitrogen ratio in the cells is not stable and it is increased in the rapidly growing tissues [14,15]. A large amount of this anion is also deposited in the bone in a fixed proportion with the calcium ion, and can act as a mineral reservoir. In fact, regardless of the proper bone metabolic status, phosphorus consumption by the cell is privileged in the growing newborn and the ion may be released into circulation from the bone if necessary for cellular requirements. When phosphorus is taken up by cells, without being adequately provided by the administered nutrition, the blood level falls, which may trigger the release of phosphorus from bone. Simultaneously, calcium mobilization results in excess calcium in the extracellular space, which can lead to hypercalcemia and hypercalcuria. Lost calcium will be found in excess in extracellular space (risk of hypercalcemia) and will stimulate its urinary excretion [21]. The strict relationship among AA, calcium and phosphorus intakes is not specific to the first days of life of parenterally fed neonates. This phenomenon of phosphorus deprivation may also be observed in preterm infants enterally fed when a supplementation of protein was not accompanied by a modification of the Ca/P ratio. The relationship between phosphorus retention, and both nitrogen and calcium retention has been previously evaluated in enteral nutrition using metabolic balance techniques [22,23].

Therefore, the association of hypokalemia, hypercalcemia and hypophosphatemia may effectively describe a new syndrome in premature infants, linked to early aggressive parenteral nutrition. However it cannot properly be considered a “re-feeding” syndrome.

**Figure 4:**
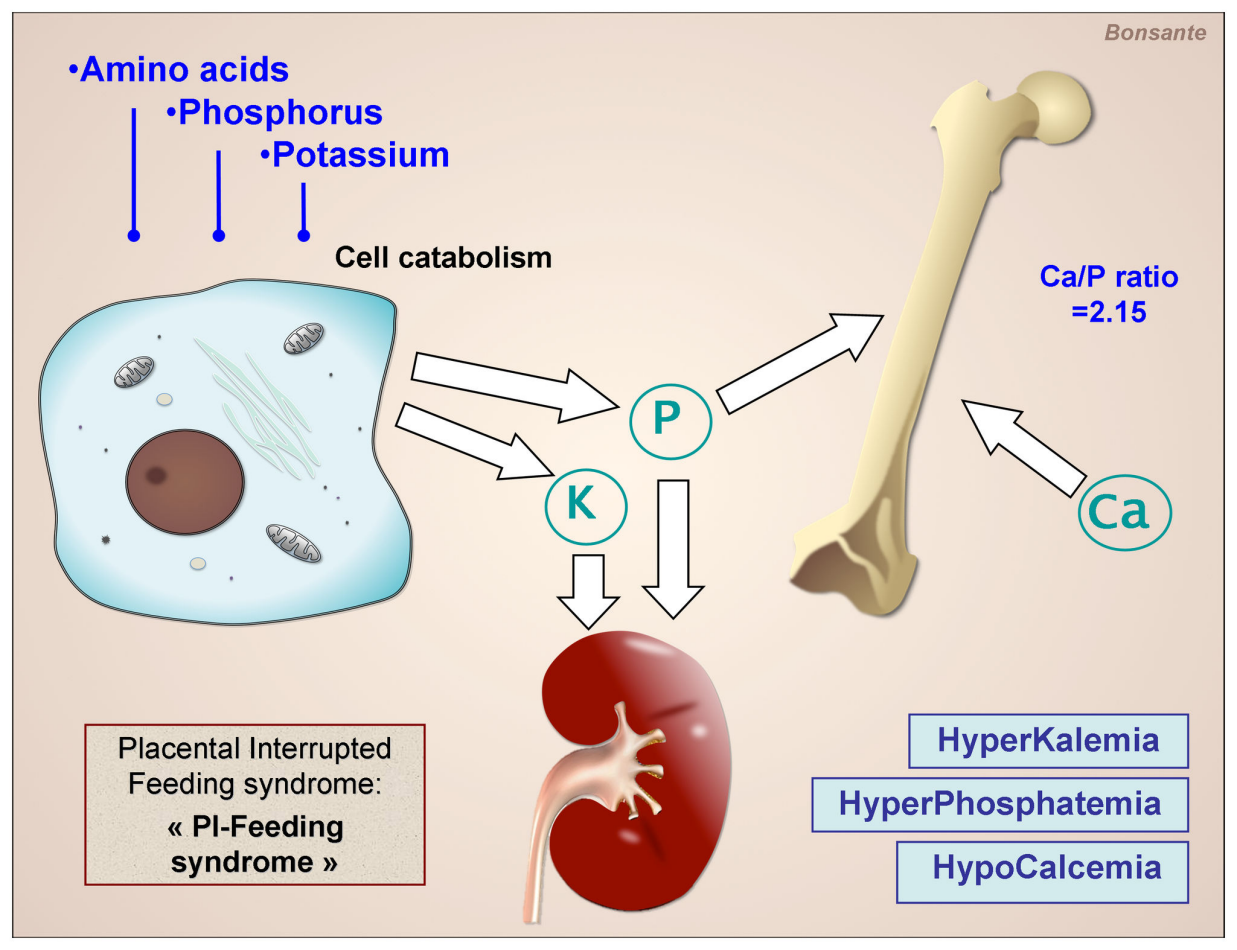
Hypothesis for the mechanism of the Placental Interrupted Feeding syndrome of the preterm infant (PI-Feeding syndrome). The abrupt interruption of the continuous placental flow of amino acids and energy promotes catabolism and ion release by the cell. This causes disturbances such as hyperphosphatemia and hyperkalemia, despite the break in phosphorus and potassium supply.

In fact, before the “early feeding era” some well known metabolic disturbances, such as hypocalcemia, hypoglycemia, non-oliguric hyperkalemia and hyperphosphatemia, reflective of the catabolic state, already represented a neonatal syndrome related to the birth and to the interruption of the continuous nutritional placental flow that we could call: Placental Interrupted Feeding syndrome of the preterm infant (**PI-Feeding syndrome**) (Figure 4).

**Figure 5:**
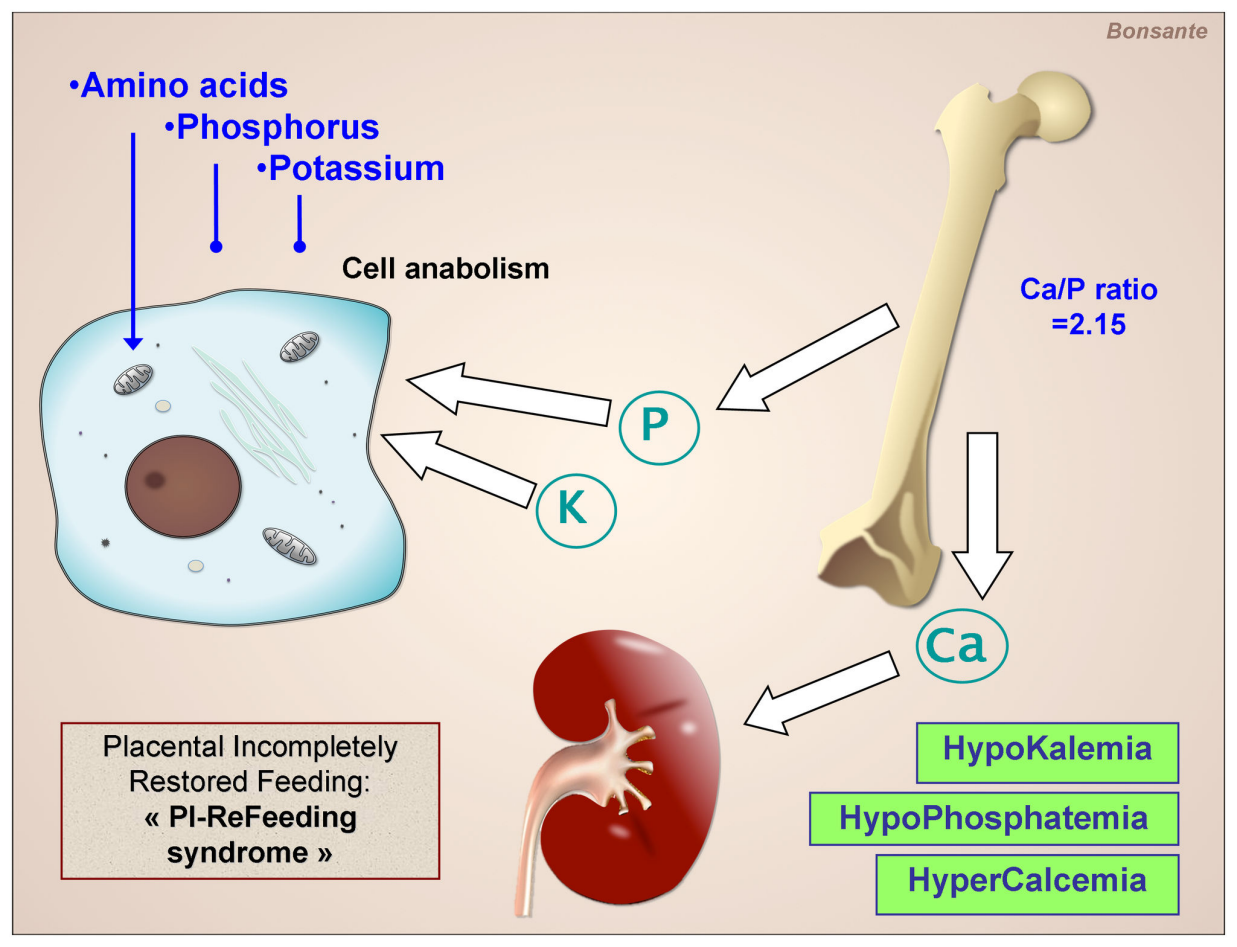
Hypothesis for the mechanism of the Placental Incompletely Restored Feeding syndrome of the preterm infant (PI-ReFeeding syndrome). The parenteral supply of amino acids and energy maintains the cell in an anabolic state and promotes its uptake of phosphorus and potassium. This causes a decrease of their plasma concentrations in the absence of an adequate intake.

The disturbances we observe now are related not to a re-feed phenomenon but to a suboptimal provision of nutrients after the Placental Feeding Disruption, since the restoration of a continuous flow of AA and energy is not accompanied by the intake of all the other nutrients. So we propose to define this condition as: Placental Incompletely Restored Feeding syndrome (**PI-ReFeeding syndrome**) (Figure 5).

### Considerations about the theoretical calculation of phosphorus needs

The attempt to calculate the ideal requirement of phosphorus in the premature infant is perhaps mission impossible. In fact, our data on specific tissue needs, on optimal bone phosphorus content, and on ideal growth pattern are incomplete; wide variations can be hypothesized according to gestational age, post-natal age, and depending on the individual variations. Furthermore, the tolerance and stability of the phosphorus in the parenteral nutrition bags limit our possibilities.

However, the phosphorus is present in tissues with somewhat fixed ratios compared to other essential constituents, allowing a correct calculation of predictable imbalances.

Our goal was therefore to estimate on the basis of defined AA and calcium intakes, whenever arbitrarily decided, the optimal supply of phosphorus, in order to minimize the risk of acute metabolic disturbances. Caution has to be exercised concerning the stability of PN solution. In our population the enteral feeding was limited during the first week of life. For this reason we elaborated the formula without considering the intestinal absorption rate of nutrients that may widely vary in the preterm infant. This has to be considered as a limitation of this study. The proposed algorithm needs to be validated in a prospective study.

## Conclusion

The present study adds more evidence to current knowledge, underlining the risk of electrolyte disturbances during early ‘aggressive’ parenteral nutrition in the preterm infant. As already observed by us and other authors, this risk of systematic perturbations may describe a syndrome due to incomplete feeding after the placenta nutrient flow disruption, the PI-ReFeeding syndrome. The effort to improve nutrition of premature infants has led many of us to focus primarily on the amount of AA and energy intake, thus in part underestimating the fundamental role of phosphorus and potassium for growth. This paper attempts a pragmatic approach to calculate the amount of phosphorus to be added in the PN bag, whilst not trying to provide the ideal intake, but rather aiming to avoid serious disturbances in the plasma concentrations of calcium and phosphate.
